# From Microbiome to Traits: Designing Synthetic Microbial Communities for Improved Crop Resiliency

**DOI:** 10.3389/fpls.2020.01179

**Published:** 2020-08-27

**Authors:** Rafael Soares Correa de Souza, Jaderson Silveira Leite Armanhi, Paulo Arruda

**Affiliations:** ^1^Centro de Biologia Molecular e Engenharia Genética, Universidade Estadual de Campinas (UNICAMP), Campinas, Brazil; ^2^Genomics for Climate Change Research Center (GCCRC), Universidade Estadual de Campinas (UNICAMP), Campinas, Brazil; ^3^Departamento de Genética e Evolução, Instituto de Biologia, Universidade Estadual de Campinas (UNICAMP), Campinas, Brazil

**Keywords:** synthetic microbial community (SynCom), plant microbiome, inoculants, metagenomics, plant growth-promoting (PGP)

## Abstract

Plants teem with microorganisms, whose tremendous diversity and role in plant–microbe interactions are being increasingly explored. Microbial communities create a functional bond with their hosts and express beneficial traits capable of enhancing plant performance. Therefore, a significant task of microbiome research has been identifying novel beneficial microbial traits that can contribute to crop productivity, particularly under adverse environmental conditions. However, although knowledge has exponentially accumulated in recent years, few novel methods regarding the process of designing inoculants for agriculture have been presented. A recently introduced approach is the use of synthetic microbial communities (SynComs), which involves applying concepts from both microbial ecology and genetics to design inoculants. Here, we discuss how to translate this rationale for delivering stable and effective inoculants for agriculture by tailoring SynComs with microorganisms possessing traits for robust colonization, prevalence throughout plant development and specific beneficial functions for plants. Computational methods, including machine learning and artificial intelligence, will leverage the approaches of screening and identifying beneficial microbes while improving the process of determining the best combination of microbes for a desired plant phenotype. We focus on recent advances that deepen our knowledge of plant–microbe interactions and critically discuss the prospect of using microbes to create SynComs capable of enhancing crop resiliency against stressful conditions.

## Introduction

In recent years, significant steps have been taken towards understanding many facets of the plant microbiome. With advances in sequencing technologies and analytical tools, we have learned that a functionally diverse microbiota is recruited from the environment and assembled into a defined structure that is dependent on soil type, host genotype and environmental changes (Walters et al., 2018; Xu L. et al., 2018; Liu et al., 2019; Zhang et al., 2019). These studies profoundly affected our perception of the complexity and dynamics of plant–microbe interactions. More importantly, they allowed the establishment of a link between microbial diversity and plant traits, such as resiliency to biotic and abiotic stresses (Gómez Expósito et al., 2017; Jacoby et al., 2017; Lemanceau et al., 2017; Compant et al., 2019). However, while the field of plant microbiome research has rapidly evolved, few if any of these novel concepts have been considered in the selection of beneficial microbes for agricultural applications.

Conventional inoculants used in current agricultural practices are generally composed of a single strain isolated by *in vitro* screening assays for plant growth-promoting (PGP) activities or inoculation experiments under controlled conditions. Despite being broadly adopted, these strategies fail to capture important aspects of plant–microbe interactions. Recent studies have shown that the plant microbiome is composed of a highly diverse and complex community, often sustained by multiple interactions between microbes and their host. Moreover, the beneficial effects of the microbiota are frequently described as being provided by synergistic interactions between microbes (Timm et al., 2016; Niu et al., 2017).

More recently, synthetic microbial communities (SynComs) have received a great deal of interest. SynComs are small consortia of microorganisms designed to mimic, at some scale, the observed function and structure of the microbiome in natural conditions. The rationale is to reduce the complexity of the microbial community while still preserving some of the original interactions between microbes and hosts, providing a repertoire of functions that would not be achievable by a single microbe (Qin et al., 2016; Niu et al., 2017; Vorholt et al., 2017; Kaminsky et al., 2019). In addition to broadening the scope of features and metabolites, SynComs may also increase community stability through synergistic interactions between their members (McCarty and Ledesma-Amaro, 2019). Notably, the major advantage of SynComs lies in the possibility of tailoring communities using concepts from microbial ecology and genetics with defined and predictable traits. In this sense, the concept of SynComs can be expanded to include the rationale of designing communities that incorporate a desired set of microbial traits for agriculture.

Tailoring SynComs has become a valuable approach for uncovering plant–microbe interactions. By adding, removing or replacing microorganisms in a SynCom formulation, the role of each microbial member can be further investigated, as well as the factors governing community assembly (Vorholt et al., 2017). In maize, for instance, removal of a single strain of *Enterobacter cloacae* dismantled a microbial community capable of reducing the severity of blight disease (Niu et al., 2017). In another example, by comparing *indica* and *japonica* rice varieties, Zhang et al. (2019) observed that the recruitment of a larger proportion of nitrogen cycle-related bacteria in *indica* was associated with NRT1.1B, a plant nitrate transporter. A SynCom containing *indica*-enriched microorganisms had a greater effect on rice growth than a *japonica*-enriched SynCom. These studies highlight that factors governing microbial community assembly should be considered when designing inoculants for agricultural applications.

Here, we argue in favor of using the SynCom concept to create consortia of microbes that can enhance plant production and resiliency against biotic and abiotic stress in agriculture. Microbiome data, such as genome and metagenome sequences, along with microbial profiling, could help design SynComs that confer stable plant phenotypes and promote robustness in terms of both plant colonization and persistence throughout plant development. We explored relevant bottlenecks, functional gaps, and underexploited tools in the plant microbiome that may help develop novel strategies for bridging microbial ecology and screening procedures associated with microbial functions towards developing microbiome technologies for agricultural sustainability.

## Identifying Relevant Microbes With Key Traits for Stable and Effective Syncoms

Traditionally, the selection of microbes for agricultural application has essentially involved *in vitro* screening for well-known taxa or PGP activities such as nitrogen fixation, phytohormone production, and 1-aminocyclopropane-1-carboxylate (ACC) deaminase activity (Glick, 2012). However, except in some extensively investigated cases, such as rhizobium–legume interactions and mycorrhizal fungi, there is still no clear correlation between these traits and their effectiveness in plant growth promotion or their contribution to sustaining stable plant–microbe associations (Finkel et al., 2017; de Souza et al., 2019). Furthermore, inoculants designed with these conventional approaches are often unable to establish and sustain associations with plants under field conditions, yielding unsatisfactory results (Nadeem et al., 2014; Zimmer et al., 2016).

Advances in microbial ecology, leveraged by high-throughput sequencing of metagenomes and molecular markers, helped to shed light onto the factors involving the successful establishment of the microbial community, as well as the reasons why some microbes used as inoculants fail to robustly colonize plants. The plant microbiome comprises highly diverse and complex microbial communities that are influenced by many factors, such as host genotype, environmental changes, and plant development (Coleman-Derr et al., 2016; de Souza et al., 2016; Liu et al., 2019). A successful inoculant must compete with indigenous microbes, efficiently colonize plant organs, and establish stable and resilient associations despite changes in the environment and soil microbial composition throughout the growing season. In this scenario, it is not surprising that common screening approaches for single traits fail to capture required traits for creating robust inoculants for applications in the field (de Souza et al., 2016; Finkel et al., 2017).

**Figure 1:**
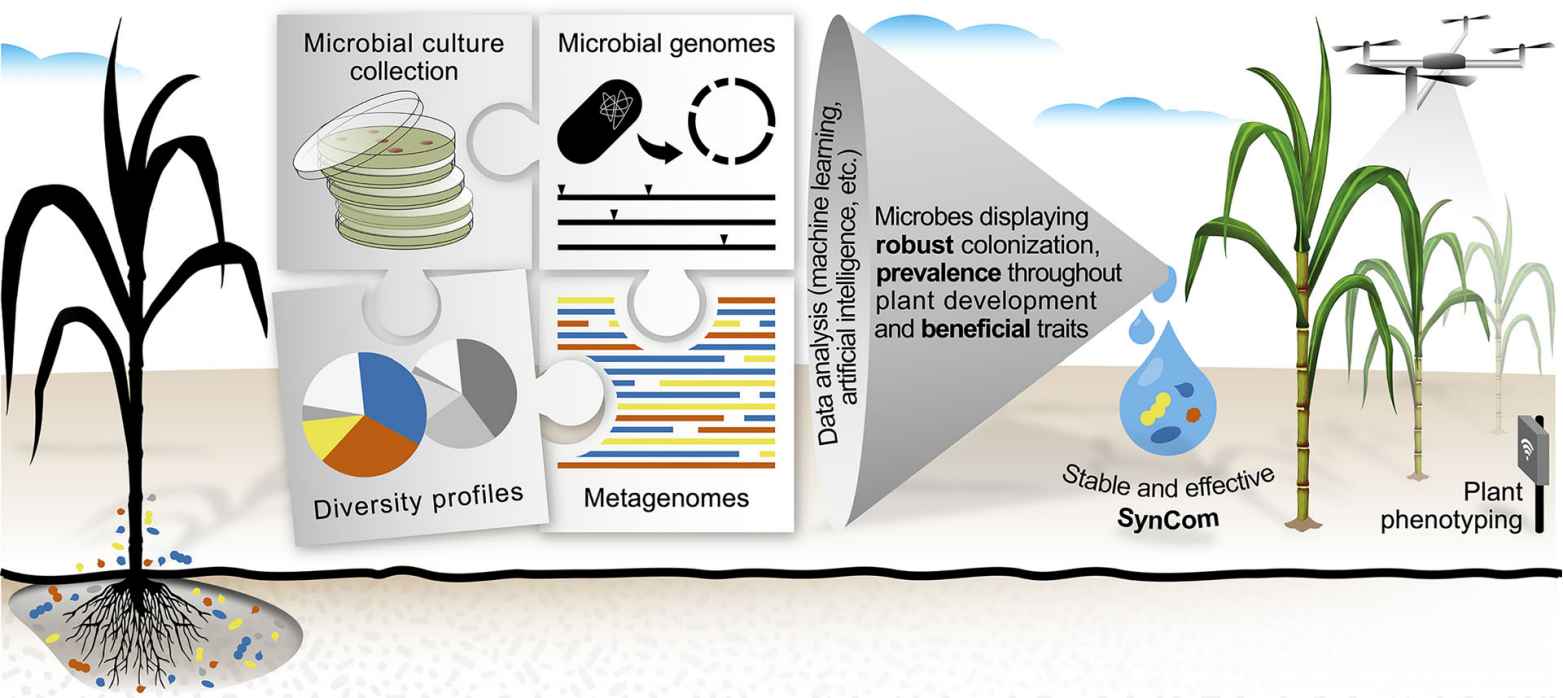
A framework for tailoring stable and effective synthetic microbial communities (SynComs) to enhance crop resiliency to environmental stresses. The selection of microbes in a culture collection is based on functional and empirical evidence, regardless of taxonomic classification. The rationale is driven by using both genome and microbial profiling data in the selection of key microbial candidates. Machine learning and artificial intelligence computational tools drive crucial steps in identifying microorganisms possessing traits for robust colonization, prevalence throughout plant development, and specific beneficial functions for plants. As a proof of concept for SynCom effectiveness, tools for plant phenotyping serve as an important diagnostic platform for measuring the impact of SynComs addressing the demand for both increased productivity and plant resiliency. The figure was prepared with the effective use of colors to help people with low visual acuity or color blindness.

Since *in vitro* evidence of such traits *per se* is insufficient to ensure that microbes are capable of eliciting the desired phenotype in plants, the incorporation of additional variables for microbial selection is highly demanded. In this context, large sequencing datasets currently available in public databases comprise a promising alternative for identifying beneficial and efficient microbes. In contrast to selecting microbes based on single PGP activities or taxonomy, genomic datasets can be used to design SynComs harboring multiple traits, such as robust colonization (high abundance in plant organs), prevalence (consistency across plant developmental stages) and specific beneficial functions (**Figure 1**).

One strategy to overcome gaps in current inoculants is to select microbes based on the diversity profile of plant microbiota. Deep-sequencing analyses of the 16S rRNA gene have revealed that certain groups of microbes are able to robustly colonize, consistently establish and sustain association with plants regardless of changes in the environment or plant developmental stages (de Souza et al., 2016; Müller et al., 2016; Xu J. et al., 2018). Members of these dominant groups, named the core microbiome, can be incorporated in SynComs, thus preventing the lack of efficiency and prevalence observed in situations where strains are outcompeted by naturally occurring microbiota. Notably, studies using both abundant and prevalent core microbes in SynComs have shown efficient colonization and beneficial effects such as plant defense against pathogens and root growth (Niu et al., 2017; Armanhi et al., 2018). These studies confirm that core microbial groups are extremely efficient at colonizing plants, highlighting that dominant groups are likely involved in functions important for plant growth and survival. Overall, incorporating both concepts into the design of SynComs is a fundamental step towards ensuring inoculum stability.

Designing SynComs containing microbes compatible with different plant genotypes and resiliency through different environments is challenging. Recent examples have shown that robust colonizing microbes from sugarcane are capable of colonizing maize and benefiting its growth (Armanhi et al., 2018). Bacterial strains isolated from lodgepole pine significantly improve maize plant biomass accumulation (Puri et al., 2015). The genome sequences of core microbiome members isolated from sugarcane shows that robust-colonizing strains are enriched in genes coding for carbon metabolism when compared with non-core microbiome strains (de Souza et al., 2019). As the genome sequences of plant microbiomes become increasingly available, comparative genomics would help to identify specific genomic markers for key traits, which will guide the selection of beneficial microbes (Finkel et al., 2017; Toju et al., 2018).

In addition to microbial profiling data, the expanding number of reference genomes and metagenomes in public databases is an important foundation for identifying microbes with desired traits. This rationale is driven by using genomic information and gene expression profiles to select microbes containing plant-beneficial functional traits or metabolic capabilities that will help in designing the best microbial combination for inoculants (Vorholt et al., 2017; Toju et al., 2018). Because important traits such as colonization efficiency and prevalence are likely associated with multiple genes, genome surveys for multiple gene markers will be key to identifying relevant microbes (Cole et al., 2017; Levy et al., 2018; de Souza et al., 2019). Ultimately, genomics-available datasets will make it easier to screen microbial candidates based on genomic markers as the use of these datasets tend to be less laborious than traditional procedures (Finkel et al., 2017). Identifying microbial candidates that contain multiple plant beneficial traits will assist to more precisely design SynComs containing microbes with synergistic traits.

In light of these massive amounts of data, computational tools such as machine learning and artificial intelligence (AI) will be critical for identifying microbial candidates from large datasets and culture collections. In biomedical science, these tools have already proven to be effective in discovering novel antibiotics (Stokes et al., 2020). In plant science, however, there are few reports employing these tools to address questions related to plant–microbe associations (Levy et al., 2018). Machine learning and AI will be critical for predicting the outcome of SynComs based on microbiomes and will likely take the field to a new level (**Figure 1**).

## Magnifying Microbial Cultivability

Building microbial culture collections is key to manipulating plant-associated microorganisms and designing SynComs with agronomic functional properties (Schlaeppi and Bulgarelli, 2015; Finkel et al., 2017; Vorholt et al., 2017). However, culture-independent data have shown that a sizeable amount of microbial diversity may remain unexplored given cultivability limitations (Lundberg et al., 2013; Turner et al., 2013). Thus, the lack of better approaches for microbe cultivation reduces our capability to design inoculants impacting plant performance and represents a major hurdle to exploring novel microbes.

Novel strategies have been proposed to maximize access to microbial strains while maintaining their viability (Kehe et al., 2019). Recently, the use of microfluidic platforms has been shown to be a promising method to cultivate hitherto-uncultured microorganisms in complex communities (Aleklett et al., 2018). By facilitating microbial interactions in a microenvironment reflective of natural conditions, microbe–microbe interactions are preserved, and microbial survival is dramatically increased (Nichols et al., 2010). In accordance, high-throughput droplet-based systems for manipulating core microbiomes have allowed the screening and isolation of sets of microorganisms based on cell sorting and encapsulation (Hosokawa et al., 2015).

Since many microbial groups required defined growing conditions, several studies have suggested different approaches to increase microbial cultivability. An example is coculturing microbial mixtures of low richness, introduced by the concept of community-based culture collection (CBC) (Armanhi et al., 2018). By picking non-confluent colonies from primary platings, regardless of whether they represent single or multiple microorganisms, this approach allows culturing communities instead of solely axenic colonies and greatly increases microbial cultivability. Instead of employing purification procedures in search of axenic cultures of all colonies, one can later target only those of interest including, for instance, robust colonizing microorganisms (Armanhi et al., 2016). Additionally, many cross-feeding compounds have already been found in microbe–microbe interactions (Kosina et al., 2016; Lubbe et al., 2017). Cocultivation empowers metabolic interactions between microbes and enables more efficient microbes to thrive. Also, simple and convenient approaches like supplementing the culture media with extracts from their environmental origin have helped to increase retrieval of microbial groups (Stewart, 2012; Armanhi et al., 2018).

Undeniably, when seeking robust colonizing microbes to compose a SynCom, the culture and identification of microorganisms have little impact unless their relevance for plants is considered. Therefore, an equally important but often neglected step is the cross-referencing of isolated microbes and the plant microbiome profile. Based on that, recent studies verified that previously obtained microbial culture collections comprise substantial proportions of the host microbiome (Bai et al., 2015; Armanhi et al., 2018). Such a strategy is built on the rationale of selecting microbes in a culture collection based on empirical evidence from microbial surveys, regardless of taxonomic classification or preselected traits (**Figure 1**). By using this approach, microbes are targeted based on relevant traits, such as robust colonization and prevalence (Armanhi et al., 2018; de Souza et al., 2019).

## Designing Syncoms for Crop Resiliency

Crop development is known to be strongly influenced by adverse environmental conditions. For instance, drought is considered one of the most severe weather events that directly reduces crop yield (Bartels and Sunkar, 2005; Yamaguchi and Blumwald, 2005). Another major constraint for crop production refers to biotic stress, which includes those caused by bacterial, fungal, and viral pathogens (Boyd et al., 2013). In addition, limited bioavailability of nutrients for plant metabolic processes is also a critical concern in arable lands, as in the case of nitrogen (Oldroyd and Dixon, 2014) and phosphorus (Sharma et al., 2013), among other macro- and micronutrients essential for crop growth.

In the last few years, a flurry of reports has supported the beneficial impact of microbes on the alleviation of detrimental effects caused by climatic events. In many of those reports, however, beneficial microorganisms were individually investigated. Notably, studies on the microbiome are gradually considering the synergistic and cumulative effects of SynComs on different microbial groups (Qin et al., 2016; Kong et al., 2018; Toju et al., 2018; Arif et al., 2020). For example, by inoculating poplar with many bacterial consortia composed of diazotrophs, Knoth et al. (2014) observed a dramatic increase in plant biomass. Additionally, very recently, Carrión et al. (2019) elegantly demonstrated that *Flavobacterium* and *Chitinophaga* together provide more consistent disease protection to sugar beet than when individually inoculated.

Under stressful environmental conditions, plants recruit sets of microorganisms with the ability to alleviate specific detrimental effects (Compant et al., 2019). This phenomenon was investigated by Naylor et al. (2017), who interestingly observed in C4 grasses under drought stress significant enrichment of Actinobacteria, a bacterial class previously reported as being related to plant growth under stress (Anwar et al., 2016). With such knowledge, it seems reasonable that traits incorporated by the SynComs should also be considered to strategically ensure plant homeostasis in unfavorable environments, thus mitigating losses in plant productivity, rather than solely targeting increases in plant performance under normal circumstances.

It is well accepted that the plant microbiome is shaped through a process of coevolution with its host under adverse environmental conditions (Theis et al., 2016). Thus, further studies have suggested that plants in stressful ecosystems may harbor microorganisms capable of electing traits of tolerance to that unfavored condition (Woodward et al., 2012; Camargo et al., 2019). A successful strategy of microbial selection aiming to further application in the field should consider microbiome origin as the first clue to microbial capabilities in an environmentally guided selection of traits. For example, microbes with a significant impact on crop stress resiliency have been isolated from saline habitats. These halotolerant bacteria are capable of mitigating salt stress in wheat (Ramadoss et al., 2013). In another significant example, bacterial strains isolated from zinc-polluted soil decreased the concentration of zinc in clover (Vivas et al., 2006).

## Assessment of Syncom Influence on Plant Productivity and Physiology

The emerging interests in designing and applying beneficial SynComs for agricultural sustainability are challenged by the demand for assessing microbial impacts on plant physiology. The assessment of plant traits conferred by SynComs requires methodologies capable of quantifying microorganisms in terms of their robustness of colonization and preferred organs in plants, as well as their capability to outcompete pathogenic resident microbiota. Such further validation should consider both adverse environmental conditions and heterogeneity in farmlands (Toju et al., 2018). Overall, beyond a deep investigation of microbial composition, the assessment of plant development and productivity is a fundamental step as a proof of concept for SynCom effectiveness. That stated, as microbes significantly affect host physiological status, phenotyping might serve as an important diagnostic platform for measuring the impact of SynComs or even detecting an imbalance in the microbiota (**Figure 1**).

Invasive and punctual approaches of plant phenotyping have conventionally been employed in a time-consuming manner. The development of automated and noninvasive techniques for measuring plants has increased for small-, medium-, and large-scale setups, especially with regard to imaging (Fahlgren et al., 2015; Rouphael et al., 2018). Optical techniques (such as RGB, infrared and hyperspectral imagery) are routinely applied in plant disease detection and crop breeding (Mahlein, 2016; Mohanty et al., 2016; Shakoor et al., 2017). As environmental parameters intrinsically face continuous fluctuations, a recent discussion pointed out the need for continuously measuring plant traits under experimental conditions (Halperin et al., 2017). Nevertheless, the lack of tools for continuous phenotyping still remains an important gap in the functional analysis of plant–microbe interactions and their application in agriculture.

Integration of phenotyping and *omics* data through machine learning and AI algorithms will be an important step towards data-driven optimization and monitoring of SynCom efficiency. By constantly monitoring and integrating multiple genomic and phenomic datasets throughout different growing seasons, the analysis platform will become increasingly robust in determining the best combination of microbes as well as predicting the outcome of SynCom inoculation. While predictive pipelines and algorithms are becoming popular, devising solutions for integrating data from different *omics* fields remains challenging.

## Conclusion and Future Perspectives

As the microbiome is extensively reported as playing fundamental roles in plant processes, the application of microorganisms in agriculture has emerged as a promising and sustainable alternative for improving crop performance, especially with regard to enhancing plant resiliency to environmental stresses. However, developing stable and effective SynComs for agriculture will require novel approaches that incorporate recent advances in microbiome research, such as the rational use of both genome and microbial profiling data in the selection of key microbial candidates.

We argued that two major factors to be considered are microbial robustness in terms of colonization and their prevalence through plant development. Identifying and incorporating robust and prevalent plant colonizers, such as those belonging to core microbiomes, has the potential to increase SynCom stability throughout the growing season and to prevent the inoculated community from being overcome by naturally occurring microbes. Additionally, the selection of microbial candidates should consider screening approaches based on the microbial genome in search of traits related to functions beneficial to plants and traits that enhance SynCom stability. A combination of these strategies will likely be leveraged by computational methods, including machine learning and AI, for the design of SynComs with predictable and successful impacts on plants.

When designing SynComs for agriculture, some constraints should also be considered. Since scaling up microbial growth in industrial processes is still a bottleneck, the ability to use a minimal number of microbes is urgently needed to reduce costs and simplify procedures, a requirement that can be achieved by tailoring SynComs whose members display synergistic and cumulative effects. The validation of SynCom stability, effectiveness and robustness of colonization is supported by sequencing techniques applied to small-scale proof-of-concept trials. Additionally, the application of SynComs to address the demand for both increased productivity and increased plant resiliency faces a further limitation regarding approaches for measuring and assessing plant performance under field conditions. Recently, efforts have been made towards developing tools capable of providing a comprehensive picture through plant phenotyping, with an emphasis on imagery. Data from such platforms will greatly contribute to quantifying SynCom efficiency and improving the SynCom design process thereafter. Although multidisciplinary approaches for integrating different dimensions of *omics* data are still lacking, the rational design of SynComs for agricultural purposes will undoubtedly create novel opportunities for sustainable production.

## Author Contributions

RdS, JA, and PA equally contributed to writing and revising the manuscript.

## Funding

We are grateful to the São Paulo Research Foundation (FAPESP) for supporting this manuscript under the project “The Genomics for Climate Change Research Center (GCCRC)”, grant 2016/23218-0, São Paulo Research Foundation (FAPESP). RdS received a postdoctoral fellowship, grant 2018/19100-9, São Paulo Research Foundation (FAPESP). JA received a postdoctoral fellowship, grant 2018/18403-8, São Paulo Research Foundation (FAPESP).

## Conflict of Interest

The authors declare that the research was conducted in the absence of any commercial or financial relationships that could be construed as a potential conflict of interest.
