# Erratum: From Microbiome to Traits: Designing Synthetic Microbial Communities for Improved Crop Resiliency

**DOI:** 10.3389/fpls.2020.614083

**Published:** 2020-11-12

**Authors:** Frontiers Production Office

**Affiliations:** Frontiers Media SA, Lausanne, Switzerland

**Keywords:** synthetic microbial community (SynCom), plant microbiome, inoculants, metagenomics, plant growth-promoting (PGP)

Due to an editorial error, the paper was incorrectly published in the *Frontiers in Plant Science* section “Plant Pathogens Interaction” instead of “Plant Symbiotic Interactions.”

The publisher apologizes for this mistake. The original article has been updated.

